# High-frequency chaotic dynamics enabled by optical phase-conjugation

**DOI:** 10.1038/srep18988

**Published:** 2016-01-07

**Authors:** Émeric Mercier, Delphine Wolfersberger, Marc Sciamanna

**Affiliations:** 1 LMOPS, OPTEL (Optics and Electronics Research Group), CentraleSupélec, Université Paris-Saclay, 57070 METZ; 2 LMOPS, OPTEL (Optics and Electronics Research Group), CentraleSupélec, Université de Lorraine, 57070 METZ.

## Abstract

Wideband chaos is of interest for applications such as random number generation or encrypted communications, which typically use optical feedback in a semiconductor laser. Here, we show that replacing conventional optical feedback with phase-conjugate feedback improves the chaos bandwidth. In the range of achievable phase-conjugate mirror reflectivities, the bandwidth increase reaches 27% when compared with feedback from a conventional mirror. Experimental measurements of the time-resolved frequency dynamics on nanosecond time-scales show that the bandwidth enhancement is related to the onset of self-pulsing solutions at harmonics of the external-cavity frequency. In the observed regime, the system follows a chaotic itinerancy among these destabilized high-frequency external-cavity modes. The recorded features are unique to phase-conjugate feedback and distinguish it from the long-standing problem of time-delayed feedback dynamics.

Dynamical instabilities due to optical feedback have been known since the early laser developments in the 1970’s^1^. While these instabilities can be inconvenient when dealing with telecommunications (which require fiber coupling where reflection may occur) or when reading optical disks, numerous studies have investigated different kinds of feedback and the dynamics they lead to: self-pulsing solutions and chaos among others^2^,^3^,^4^. These studies allowed for the development of applications using chaos in semiconductor lasers with feedback, including random number generation^5^ and encrypted communications^6^,^7^.

Random number generators need a fast entropy growth, and encryption must have a high data rate, meaning that these applications require high frequencies and large bandwidths to operate^8^,^9^. The security of the encryption also relies on chaos complexity and the robustness of synchronization. Time-delayed feedback in a semiconductor laser generates chaos that have these properties. Nonlinear frequency mixing leads to high harmonics of the external-cavity frequency^9^ and large bandwidths that can exceed the frequency of relaxation oscillations of the laser thanks to various enhancement techniques^9^,^10^,^11^. The time-delayed nature of the system also ensures a chaos of high dimension, with a large number of Lyapunov exponents^12^.

The main technique for achieving feedback in a semiconductor laser is called conventional optical feedback (COF) and simply consists in adding a mirror to reflect part of the emitted beam back into the laser cavity. The field then interacts with its time-delayed value, the time-delay being fixed by the external-cavity length. This technique has been extensively studied and is used in numerous schemes designed for encrypted communications^4^,^7^,^9^,^10^,^11^,^13^. In this paper, we focus on an alternative and less-studied technique: phase-conjugate feedback (PCF) which consists of feeding the phase-conjugate of the emitted beam back into the laser cavity. Similarities exist between COF and PCF^14^, but we will show here that we can extract a chaotic signal with a wider bandwidth from the PCF configuration. In the range of feedback strengths achieved with the phase-conjugate mirror, we observe a chaos bandwidth enhancement of up to 27%.

By using a large sampling-rate to acquire time traces, we can implement a new measurement technique: short-time Fourier transform (STFT). With this technique, we will show that the bandwidth enhancement can be attributed to the onset of destabilized self-pulsing modes^15^, called external-cavity modes (ECMs), which go up in frequency as we increase the reflectivity of our phase-conjugate mirror^16^,^17^,^18^. The short-time frequency analysis reveals unique features of PCF that contrast with COF and therefore overcome the traditional knowledge of dynamics induced by time-delayed feedback. Although not demonstrated explicitly here, the possibility to achieve larger bandwidths by simply replacing conventional by phase-conjugate feedback suggests an improvement of performances for chaos-based random number generation.

## Results

### Experimental setup

**Figure 1:**
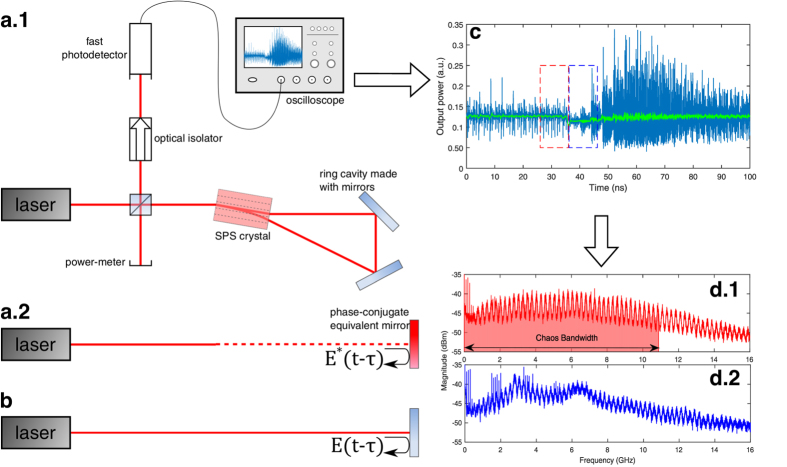
High-frequency dynamics of a laser diode with optical feedback. (**a.1**) The experimental setup and (**a.2**) the equivalent phase-conjugate mirror. (**b**) The second external cavity used to compare conventional optical feedback with phase-conjugate feedback. (**c**) High-frequency chaotic dynamics. (**d**) Post-processing analysis reveals frequency content of dynamics before (**d.1**) and after (**d.2**) power dropouts. The chaos bandwidth measured for (**d.1**) is highlighted in red.

The experimental setup is composed of an edge-emitting near-infrared laser diode whose wavelength is *λ* = 852 nm, and injection current *J* ranges from 25 to 35 mA (threshold is *J*_0_ = 14 mA). It is subjected to either PCF or COF with the intention of comparing the two configurations (see Fig. 1).

Phase-conjugate feedback is achieved through self-pumped four-wave mixing in a photorefractive *Sn*_2_*P*_2_*S*_6_ (SPS) crystal, in a ring cavity configuration^19^, as presented in Fig. 1a.1. The wave mixing between the incident beam and the beam fanning creates a transmission refractive-index grating in the crystal. By propagating through this grating, the transmitted beam becomes phase-conjugated. The actual distance that light travels before going back into the laser cavity thus corresponds to twice the distance between the laser and the crystal, plus twice the optical path length of the crystal, plus the length of the ring cavity^20^, which corresponds to the equivalent setup presented in Fig. 1a.2. To compare with COF, we simply put a conventional mirror in place of the equivalent phase-conjugate mirror so that the effective external-cavity length is the same (see Fig. 1b). In our study, we adjust the optical length of the external cavity to *L* = 119 cm (120 cm for COF), corresponding to a time delay 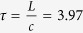 ns and an external-cavity frequency 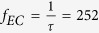 MHz. The reflectivity *R* of this equivalent phase-conjugate mirror can be varied by inserting a variable density filter along the beam path. The range of reflectivities that can be attained is 0–14% in the case of PCF and 0–90% in the case of COF. PCF ensures a highly efficient coupling of the feedback field in the laser cavity, thanks to its self-aligning property. By contrast, COF requires a fine adjustment of the feedback coupling efficiency. We have optimized the COF coupling efficiency by maximizing the current reduction of the laser diode threshold^21^. The feedback ratio or what we call the reflectivity R is then adjusted with a variable attenuator placed in the feedback path.

Via a beam splitter inserted in the path of the output beam, part of the power is launched in the measurement arm, which consists of an optical isolator and a fiber coupler. The fiber is then coupled to a 12 GHz photodetector (NewFocus 1554-B), itself connected to a fast oscilloscope acquiring data at the rate of 100 GSa.s^−1^ with a 16 GHz bandwidth (Tektronix DPO 71604C). The large sampling-rate over a large bandwidth allowed us to develop new specific techniques for analyzing the high-frequency dynamics including the short-time Fourier transform (STFT), as detailed below. The beam splitter also allows us to measure the power returning to the laser and thus to calculate the reflectivity of the mirror, knowing the power emitted by the laser at a given injection current.

### Analysis of chaotic dynamics

We choose parameters so that the laser is destabilized into a well-known dynamical regime called low-frequency fluctuations (LFF)^1^,^22^,^23^. In this regime, the output power of the laser is chaotic and power dropouts occur randomly. This dynamics is explained by an itinerancy among destabilized external-cavity modes (ECMs)^15^,^24^. Dropouts occur when the trajectory of the system is repelled from a stable ECM. It is important to note that ECMs are self-pulsing states oscillating at multiples of *f*_*EC*_ in PCF^16^,^18^, whereas they are stationary states in COF^24^. This distinction will become essential in the following analysis of the high-frequency dynamics. A typical trace is shown in Fig. 1c in light blue. Superimposed in green is the low-pass filtered trace that allows us to better identify the power dropouts.

The purpose of the following analysis will be to gain insight into the frequency dynamics underlying LFF both in PCF and COF. To that end, we wish to discriminate the frequency content before the dropout and just after a dropout. Therefore, we developed a concatenation technique: for each dropout in the time traces we acquire, we extract the 10 ns before the dropout (red dotted rectangle in Fig. 1c), concatenate them together and compute the fast Fourier transform of the concatenated trace to obtain the red spectrum shown in Fig. 1d.1. We use the same technique for the 10 ns following the dropout (blue dotted rectangle) and obtain the blue spectra in Fig. 1d.2. The downside of this technique is that it introduces concatenating artifacts in the spectra at multiples of 0.1 ns^−1^. But these artificial peaks are negligible because they are very narrow, usually no more than one point among the thousands of points that compose the total spectrum.

### Chaos bandwidth

Such time-resolved spectra will be further used to evaluate the bandwidth of the system. The bandwidth is defined as the frequency that contains 80% of the total power of the spectra, as commonly used in the literature^10^,^11^. For the case of Fig. 1d.1, the bandwidth extends up to about 10.9 GHz which is the upper bound of the red area underlying the spectrum. The concatenating artifacts mentioned earlier would influence our results here but as already stated, they are negligible because they represent only a few points. And even if they had an influence, they would lower the bandwidth since they are present at low frequencies. In addition, we also calculated the spectra of the 10 ns for each dropout individually and then averaged them to obtain the mean spectrum. The resulting averaged spectra yielded conclusions similar to the ones obtained with the concatenation technique.

**Figure 2:**
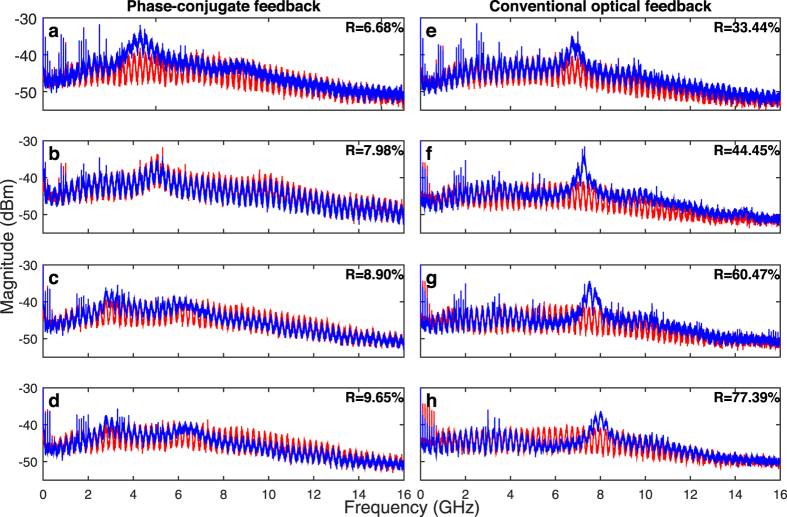
rf spectra computed with the concatenation technique on time traces showing LFF while increasing *R* from top to bottom. (**a**–**d**) (**e**–**h**) show results for PCF (COF). Values of reflectivity *R* are reported on the corresponding figures. Injection current *J* was set to 30 mA.

The concatenation technique is used to compute the spectra in Fig. 2. We observe that oscillations after the dropout are dominated by the relaxation-oscillation frequency *f*_*RO*_^22^, accompanied by some period-doubling with 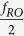 resonating, especially in the case of PCF (Fig. 2a–d). From the literature^25^, we know that increasing the reflectivity can shift *f*_*RO*_ toward higher frequencies: *f*_*RO*_ goes from 4.5 to 6.5 GHz when increasing *R* from 6.68% to 9.65% in the case of PCF, and from 7 to 8 GHz in the case of COF when increasing *R* from 33.44% to 77.39%.

The rf spectra *after* dropouts are similar when comparing PCF with COF. However, the frequency content *before* dropouts is quite different. In the case of PCF, the spectrum extends up to higher frequencies than in COF. Also, the high frequency content increases with *R*, whereas in the case of COF, the frequency content before dropouts remains relatively independent from *R*.

**Figure 3:**
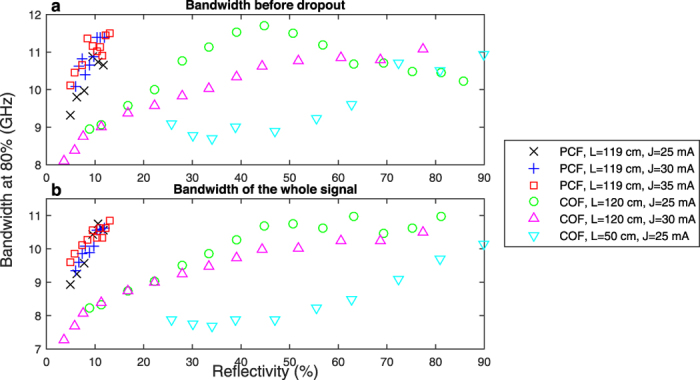
Increasing the bandwidth of a chaotic laser diode with PCF. Evolution of the bandwidths, in different configurations, for (**a**) the dynamics before dropouts and (**b**) the whole dynamics.

To illustrate this conclusion, we compute the bandwidths of the spectra before dropouts and report them in Fig. 3a. We observe that over the course of a few % of reflectivity increase, the bandwidth before dropouts increases drastically in the case of PCF, whereas in the case of COF, to observe the same bandwidth improvement, we have to increase *R* from 20 to 80% (comparing PCF with COF at *J* = 30 mA). In the range of reflectivities where comparison holds, i.e. being limited to about 14% due to the limited nonlinear interaction strength of the phase conjugate configuration, the bandwidth enhancement ranges from 21% at 5.9% reflectivity to 27% at 12.1% reflectivity.

Interestingly, when comparing the evolution of bandwidth before dropouts (Fig. 3a) and the evolution of bandwidth of the whole signal (Fig. 3b) in the two different configurations, we see that they follow the same trends, with almost the same values. From this result we can conclude that the total bandwidth of the chaotic dynamics is correlated with the bandwidth of the dynamics occuring before the power dropouts. In the next section, we will focus on the analysis of time-resolved frequency dynamics to understand how PCF differs from COF and to find why we unlock higher bandwidth with PCF.

For the sake of completeness, we also compare the bandwidth with the one achieved when COF occurs at the position of the crystal, i.e. with a shorter cavity. The results show that the bandwidth is much lower than in the case of the longer external cavity, i.e. the bandwidth increases with the time-delay. We could not test a shorter cavity in the case of PCF because of the way phase-conjugation is made, we cannot get rid of the length of the cavity ring, and the crystal was already at the nearest possible position from the laser diode.

### Time-resolved frequency dynamics

**Figure 4:**
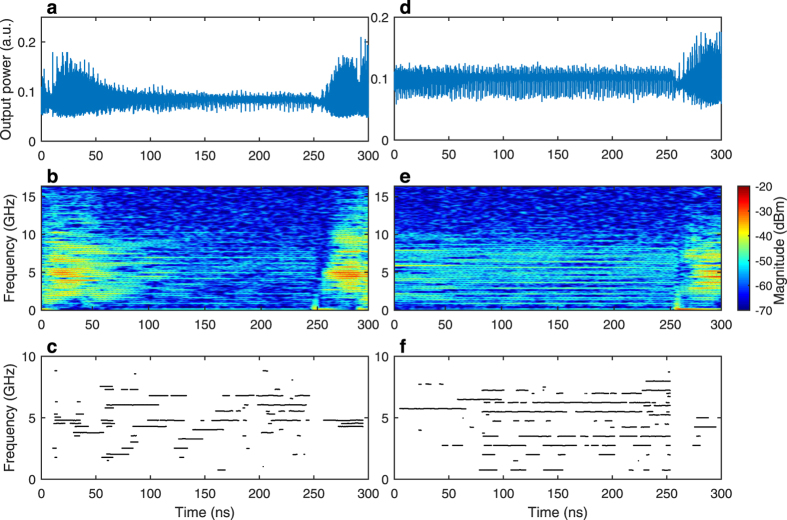
Evolution of dynamics as time passes in PCF (left column) and COF (right column). (**a**,**c**) Time traces acquired at 100 GSa.s^−1^ and (**b**,**d**) corresponding spectrograms computed with STFT. (**c**,**f**) Frequencies of the dominant modes versus time (see text for details).

The previous analysis suggests to get a closer look into the frequency dynamics on short time scales, especially before a dropout. For that purpose we apply a short-time Fourier transform (STFT) analysis on samples from the time traces. STFT consists of computing a fast Fourier transform on a small window of a signal (we use a 10 ns window, the same value that was used for the concatenation technique), then shifting this window to the next portion of the signal, and repeating the process to scan the entire time trace. Overlapping between consecutive windows (here taken as 95%) and zero-padding are used to improve the time and frequency resolutions of the resulting spectrograms. Figure 4 only shows snapshots of time traces but we observed a similar behavior when making a systematic analysis of power dropouts for several values of experimental parameters (*J* and *R*).

Figure 4 shows the evolution of the output power of the laser versus time (a,d), the associated spectrogram (b,e) and the evolution of the dominant frequencies versus time (c,f) in PCF and COF configurations respectively. Dominants frequencies are those that share the frequency with the maximum power, within a 2 dB tolerance. Just after a dropout, i.e. for the first and last ns of the sample shown in Fig. 4c, the dynamics is dominated by frequency components around the relaxation oscillation frequency. However, before a dropout, the frequency content is always changing, with specific frequencies close to harmonics of *f*_*EC*_ starting or ending to resonate. Especially, Fig. 4c indicates that there is a tendency for the system to build up higher and higher frequencies as time goes on. For example, at 135 ns the dominant frequencies are around 3–4 GHz, and a few nanoseconds later at 175 ns, dominant frequencies are around 5–7 GHz. This erratic onset of harmonic frequencies of the external-cavity frequency with a directional motion in favor of high frequencies is in good agreement with the theoretical predictions in^15^ where in the LFF regime, the system is predicted to have a chaotic itinerancy among the ruins of self-pulsing ECMs, each with their own frequency being a multiple of *f*_*EC*_. This fast evolution of the *electrical* frequency should not be mistaken with the self-scanning of the *optical* frequency reported in experiments with PCF^26^,^27^. The self-induced frequency scanning in PCF experiments is typically related to moving gratings in the photorefractive crystal and occurs anyway on a much longer time-scale (several seconds) than the time-scale of our experiment (nanoseconds). Measuring the evolution of the optical spectrum at a nanosecond time-scale would not be possible in our experiment. However, we measured the evolution at a slow rate with a confocal Fabry-Perot interferometer and we did not observe this frequency self-scanning in the chaotic dynamics of Fig. 4.

In the case of COF, shown in Fig. 4 too, the behavior of the system after a dropout is similar to the PCF case, with frequencies around *f*_*RO*_ being dominant. However, before a dropout, we observe (Fig. 4f) simultaneous resonance of frequency components close to multiples of *f*_*EC*_, which is significantly different from the erratic behavior observed in the PCF case. LFF in COF have been described as a chaotic itinerancy with a drift^22^,^24^ which excites destabilized steady-states. All of these destabilized modes have a similar frequency signature which is non-linear frequency mixing between *f*_*RO*_ and sidebands at multiples of *f*_*EC*_. Therefore when the system jumps from the ruin of a mode to the next one, the frequency spectrum does not vary much.

The difference in the manifestation of itinerancy observed with the spectrograms therefore lies in a difference in the physics responsible for the onset of ECMs. ECMs in PCF are self-pulsing solutions that, when destabilized, yield high-frequency components at harmonics of the external-cavity frequency. By contrast, ECMs in COF are steady-state solutions, that when destabilized share a similar frequency content. Therefore, using PCF unlocks spectral components of higher frequencies which then lead to the observed increase in bandwidth.

## Discussion

In summary, we have investigated chaotic behavior (LFF regime) of a semiconductor laser subjected to PCF and COF in a long cavity with moderate to strong feedback strength. We have shown that the bandwidth of a system with optical feedback can be improved by using PCF instead of COF. We have attributed this bandwidth improvement mainly to the high-frequency dynamics that take place before dropouts. Finally, we have clarified the occurrence of pulsing at harmonic frequencies of the external-cavity frequency in PCF, in agreement with recent theoretical analysis of LFF in PCF^15^.

As evidenced here and besides its fundamental interest, the physics underlying phase-conjugate feedback dynamics yields higher frequency chaotic dynamics than the one achieved with the traditional optical feedback configuration. Still, the present experiment does not aim at setting a new record on optical chaos bandwdith. Optical chaos extending up to 26,5 GHz bandwidth has for example be achieved by injecting the chaos light from a DFB laser with conventional feedback into a fiber ring resonator^10^. The bandwidth is however not related to the physics of the laser diode with feedback but to the tuning of the ring resonator. It is also known that schemes using optical injection can further extend the chaos bandwidth up to 20 GHz^9^ and even further (32,3 GHz) using dual wavelength injection^11^. Injection schemes require however a careful matching of the coupled laser multiple parameters. These and similar bandwidth enhancement techniques^28^,^29^ can also be applied to further optimize the chaos bandwidth achieved here with phase-conjugate feedback.

Additionally, we have shown here that the improvement of bandwidth lies in the self-pulsing nature of the ECMs in PCF. And since adjusting the external-cavity length and the reflectivity have an impact on the frequencies of the ECMs^16^,^17^,^18^, one reasonably expects that obtaining a larger range of reflectivities could also improve the bandwidth of our system. Other configurations for the phase-conjugate mirror exist, using total internal reflection in the crystal^19^, and could lead to higher values of the reflectivity.

Finally, although the main focus here is on the fundamental properties of phase-conjugate feedback dynamics, we believe this work motivates research in other directions. Since sequences of chaotic high-frequency pulses at harmonic frequencies appear in the recorded chaotic time-trace - due to onset of the destabilized ECMs of the PCF laser system- it would be interesting to check if this property reflects itself into the spectrum of the Lyapunov exponents and the resulting entropy growth for random number generation^4^,^5^. Of particular interest is to check whether phase-conjugate feedback would improve the entropy growth rate when compared with conventional optical feedback, besides its impact on the chaos bandwidth.

## Additional Information

**How to cite this article**: Mercier, E. *et al.* High-frequency chaotic dynamics enabled by optical phase-conjugation. *Sci. Rep.* **6**, 18988; doi: 10.1038/srep18988 (2016).
